# Pulmonary pleomorphic carcinoma with pembrolizumab monotherapy

**DOI:** 10.1002/rcr2.597

**Published:** 2020-06-12

**Authors:** Hao‐Chun Chang, Chia‐Lin Hsu, Yih‐Leong Chang, Chong‐Jen Yu

**Affiliations:** ^1^ Division of Pulmonary and Critical Care Medicine, Department of Internal Medicine National Taiwan University Hospital Taipei Taiwan; ^2^ Department of Pathology National Taiwan University Cancer Center and National Taiwan University Hospital Taipei Taiwan

**Keywords:** Lung cancer, pembrolizumab, pleomorphic carcinoma

## Abstract

Adenocarcinoma (ADC) is the most common form of lung cancer; however, some other types of lung cancer can sometimes mimic ADC. It then takes experienced pathologists and special stains to make the correct diagnosis. Here, we present a patient with pulmonary pleomorphic carcinoma who was misdiagnosed with ADC, and was treated with pembrolizumab for 13 months. The diagnosis and treatment (including immunotherapy) are also reviewed.

## Graphical abstract

Adenocarcinoma (ADC) is the most common form of lung cancer; however, some other types of lung cancer, such as pulmonary pleomorphic carcinoma (PC), can sometimes mimic ADC. Here, we present a patient with initial diagnosis of pulmonary ADC later diagnosed with PC.

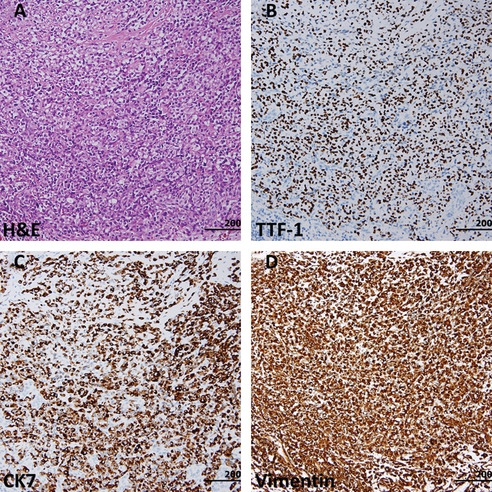

## Introduction

Pulmonary adenocarcinoma (ADC) is the most common lung cancer in Taiwan [1]. There are many treatment options for advanced pulmonary ADC, including immunotherapy, targeted therapy, and chemotherapy [2]. However, drug resistance usually occurs, despite the adequate therapy having been chosen. Multiple mechanisms of drug resistance were studied, including acquired new driver mutation and cellular transformation [3, 4, 5, 6]. Moreover, some other types of lung cancer may mimic ADC and have different prognosis, including pleomorphic carcinoma (PC), combined small cell lung cancer (SCLC), and ADC with neuroendocrine differentiation (NED). All of them can have ADC in the majority of the tumours and need careful examinations by the pathologists plus special stains to differentiate [7]. Here, we present a patient with initial diagnosis of pulmonary ADC later diagnosed with PC.

## Case Report

Our patient was a 48‐year‐old female with no previous systemic disease. She was an active smoker with 0.5 pack per day for 10 years. Productive cough with yellowish sputum and exertional dyspnoea occurred in March 2018, at age 46. She visited a hospital where chest X‐ray (CXR) revealed massive left pleural effusion. Admission was arranged and a pig‐tail catheter was inserted for pleural effusion drainage. Chest computed tomography (CT) scan was done and found a 1.76‐cm left upper lobe (LUL) lung tumour, close to great vessels, and pleural seeding. Brain magnetic resonance imaging (MRI) scan revealed multiple small brain metastases. Whole‐body bone scan showed no bony metastasis. Video‐assisted thoracoscopic surgery (VATS) of left pleura wedge resection biopsy was performed, and the pathology reported ADC, with positive thyroid transcription factor‐1 (TTF‐1), wild‐type epidermal growth factor receptor (EGFR) mutation, negative anaplastic lymphoma kinase (ALK), and >80% of programmed death‐ligand 1 (PD‐L1) expression. With the diagnosis of stage IVB (cT4N1M1c) pulmonary ADC, she visited our pulmonology outpatient clinic for a second opinion.

After discussion, tri‐weekly pembrolizumab (2 mg/kg) monotherapy was commenced from May 2018 considering the high percentage (>50%) of PD‐L1 expression in the tumour. Cyber knife stereotactic radiosurgery to the brain metastases was performed. The best tumour response was partial remission (PR) based on CT assessment every three months. A positron emission tomography (PET) scan in January 2019 disclosed suspicious malignancies at LUL of lung, and aortocaval and right iliac lymph nodes. Radiotherapy was given to LUL residual tumour and those lymph nodes. After 16 cycles of pembrolizumab treatment, brain MRI scan in June 2019 showed enlarged left parietal metastasis and chest CT scan found new right paratracheal lymph nodes, while the original LUL tumour had disappeared. Endobronchial ultrasonography (EBUS) transbronchial needle aspiration (TBNA) was arranged to the group 4R lymphadenopathy (LAP). The pathology report revealed metastatic carcinoma, with diffusely positive for cytokeratin (CK) and negative for TTF‐1, while the tumour cells were pleomorphic with large nuclei, prominent nucleoli, and frequent mitoses. Due to prolonged menstruation and abdominal throbbing pain, which would extend to her back and right thigh with right leg weakness since March 2019, she visited a gynaecologist. A transvaginal sonography (TVS) in June 2019 noted multiple uterine myomas. Total abdominal hysterectomy and bilateral salpingo‐oophorectomy were performed in July 2019 and the pathology showed no malignancy. Pemetrexed (500 mg/m^2^) + carboplatin (area under the curve (AUC) = 5) every three weeks were started later in August 2019.

**Figure 1:**
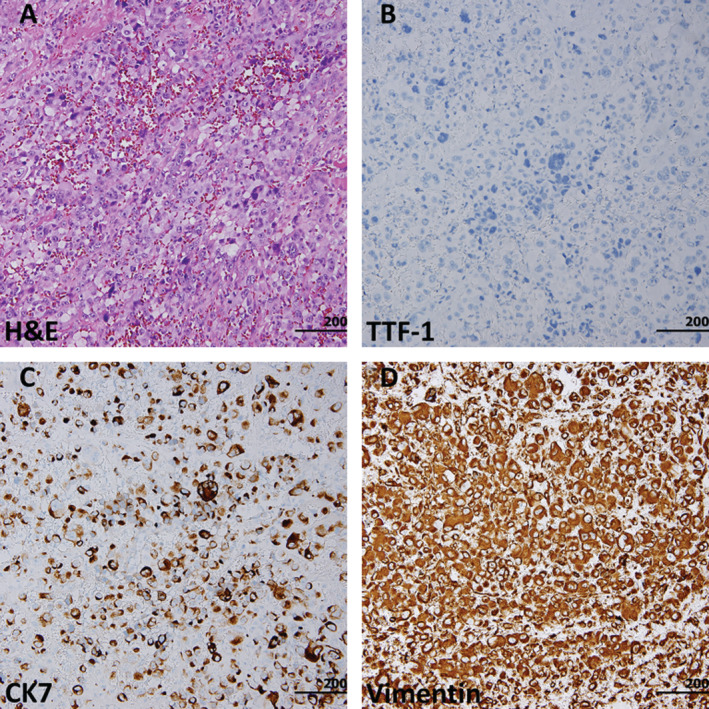
The cells of the right thigh tumour were round to polygonal with large or pleomorphic nuclei, arranged in sheets and involving the skeletal muscle (A). They showed thyroid transcription factor‐1 (TTF‐1) negativity (B), with co‐expression of cytokeratin (C) and vimentin (D).

However, exertional dyspnoea, dysuria, and general malaise occurred in September 2019. She visited emergency room, where mild fever (37.6°C), leucocytosis (white blood cell (WBC) = 33.76 K/μL), normocytic anaemia (haemoglobin (HB) = 6.4 g/dL), and microhaematuria (urine red blood cell (RBC) = >100 high‐power field (HPF) and mild pyuria (urine WBC = 10‐19 HPF)) were noticed. Blood transfusion was given while Tazocin (piperacillin + tazobactam, Pfizer Inc., U.S.A.) was administered for suspecting urinary tract infection. She was admitted. Progressive right thigh swelling and pain were noticed. Whole‐body CT scan revealed some ascites; multiple LAP at mediastinum, retrocrural, para‐aortic, paracaval, mesenteric root, bilateral iliac, and inguinal regions; as well as tumours at urinary bladder, right psoas muscle, right retroperitoneum, right buttock, and bilateral thighs. Right thigh fasciotomy and myomectomy were performed. Massive haematoma with muscle necrosis was noted at anterior thigh during the operation, and the pathology showed pleomorphic tumour cells with pale eosinophilic cytoplasm, large and vesicular nuclei, frequent mitoses, positive CK, and negative TTF‐1, which was compatible with PC (Fig. 1). Furthermore, tumour bleeding with persistent anaemia was noted after the surgery, despite frequent blood transfusion. Progressive renal function deterioration with eventually anuria and refractory hyperkalaemia occurred. She passed away with disseminated intravascular coagulation, severe lactic acidosis, profound shock, and respiratory and renal failure, 18 months after diagnosis.

**Figure 2:**
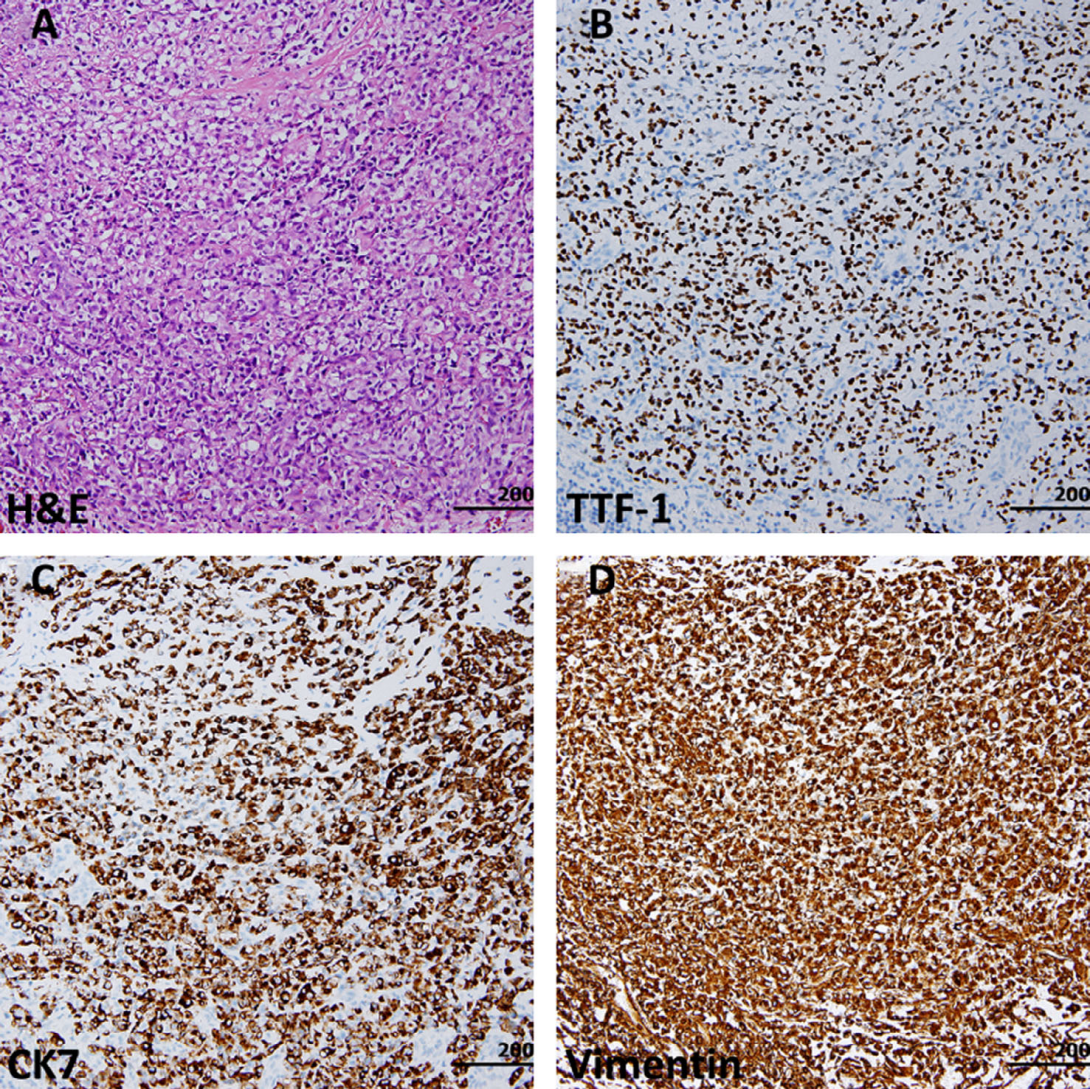
The pleural tumour was composed of epithelioid cells in solid sheets. No tumour nests, representative of epithelial differentiation, were noted (A). The tumour cells were immunoreactive to thyroid transcription factor‐1 (TTF‐1) (B), cytokeratin (CK) 7 (C), and vimentin (D).

As both the second and third biopsy results showed diffuse pleomorphic cells, we requested the pathologist to review the specimens of her first biopsy (VATS wedge resection) from the first hospital. There were solid sheets of epithelioid tumour cells in the subpleural nodules. No glandular structures or epithelial nests representative of epithelial differentiation were noted. The tumour cells were immunoreactive to TTF‐1 and showed co‐expression of CK7 and vimentin (Fig. 2), indicating sarcomatous transformation of an ADC.

## Discussion

We herein reported a patient with initial diagnosis of pulmonary ADC. However, after careful review by the pathologists, PC should be the diagnosis throughout the course. The original tissue showed both epithelial and sarcomatous features, with positive TTF‐1, CK7, and vimentin. After 16 cycles of pembrolizumab therapy, at the length of 13 months, the tissue turned into sarcomatous component only (negative TTF‐1 and CK7 with positive vimentin). Pemetrexed + carboplatin was administered as a rescue therapy, but the effect was limited. Multiple metastases were noted and our patient passed away of multiple organ failure.

According to the 2015 World Health Organization (WHO) classification of lung tumours, pulmonary sarcomatoid carcinoma is a group of cancer consisting of five different subtypes, including PC, spindle cell carcinoma, giant cell carcinoma, carcinosarcoma, and pulmonary blastoma [7]. These tumours are rare and account for less than 1% of all non‐SCLC (NSCLC), with worse outcome than other cell types [8]. Immunohistochemically, pulmonary sarcomatoid carcinoma typically demonstrates decreased or absent expression of CK, CK7, and TTF‐1 [9]. PC is a poorly differentiated NSCLC containing at least 10% of spindle and/or giant cells on the base of squamous cell carcinoma, ADC, or undifferentiated NSCLC, or a carcinoma consisting only of spindle and giant cells [10]. In other words, a tumour consisting 90% of ADC, while the other 10% are spindle or giant cells, can be defined as PC. The definition for combined SCLC and combined large cell neuroendocrine carcinoma (LCNEC) is a tumour consisting different types of lung cancer cells, with at least one of them being SCLC or LCNEC. There is no regulation for the proportion of the cell types, which means that even when 99% of the tumour is ADC but 1% of it is SCLC, it is still defined as combined SCLC, instead of ADC. Therefore, to make the correct diagnosis, it takes careful examinations by experienced pathologists, well acquainted with lung cancer classification. Even if the appearance of the specimen seems like ADC, immunohistochemical stains are needed. CK/CK7 and TTF‐1 are used to confirm pulmonary origin, while vimentin is used for sarcomatous feature and synaptophysin, chromogranin, and CD56 are markers for NED. Without incorporating these markers, the diagnosis can be erroneously made as ADC [7].

For pulmonary PC, men are affected more often than women, and it is associated with smoking [11]. The overall survival for advanced, inoperable patients is poor, usually under eight months [11, 12, 13, 14]. The standard chemotherapy regimen for NSCLC is platinum doublet, but it has limited effects for PC [13, 14]. KRAS, MET, and EGFR mutations can be found in these tumours and have the potential to be treatment targets. However, these mutations can only be detected in about 20% of patients [15, 16, 17, 18, 19]. PD‐L1 is a marker that can guide immunotherapy. Studies have shown that more than 70% of PC have PD‐L1 expression [20, 21], and treatments with pembrolizumab and nivolumab can be effective [22, 23, 24]. However, some studies are suggesting that PD‐L1 expression is higher in the sarcomatous part than in the carcinomatous area [25] and is related to more rapid progression, indicating a worse prognosis [26, 27].

PC can be easily misdiagnosed as other subtypes of NSCLC if the sarcomatous proportion is low or the pathologist does not keep an alternative diagnosis in mind. Despite studies having been so far only limited to case reports, immunotherapy (pembrolizumab and nivolumab) is an effective treatment option guided by PD‐L1 expression.

### Disclosure Statement

Appropriate written informed consent was obtained for publication of this case report and accompanying images.
